# Infectious endophthalmitis at a Philippine tertiary hospital: a ten-year retrospective study

**DOI:** 10.1186/s12348-020-00208-0

**Published:** 2020-08-14

**Authors:** Victoria Grace Dimacali, Ruben Lim Bon Siong

**Affiliations:** Department of Ophthalmology and Visual Sciences, College of Medicine-Philippine General Hospital, University of the Philippines Manila, Manila, Philippines

**Keywords:** Endophthalmitis, Infection, Trauma, Exogenous, Endogenous, Post-operative, Keratitis, Bleb, Suture

## Abstract

**Background:**

Endophthalmitis is a sight-threatening disease characterized by marked progressive inflammation of the vitreous and/or aqueous humors. Limited information is currently available regarding endophthalmitis in the Philippines. This study aimed to provide long-term summary data on endophthalmitis cases seen at the largest tertiary referral hospital in the Philippines.

**Methods:**

All endophthalmitis cases diagnosed at the Philippine General Hospital from January 1, 2007 to December 31, 2016 were retrieved and classified by etiology. Data pertaining to history, associated risk factors, isolated microorganisms, management, and final visual outcomes for each patient were reviewed.

**Results:**

A total of 202 cases diagnosed within the audit period were included in the study. These were classified as post-traumatic (55.94%), endogenous (14.36%), acute post-operative (10.40%), keratitis-induced (6.93%), chronic post-operative (5.44%), bleb-associated (3.96%), and suture-associated (2.97%) endophthalmitis. Males comprised 71.29% of the population, while the largest age group affected was 0–10 years (24.75%). The culture-positive rate was 57.89%. The predominant etiology was Gram-positive bacteria (38.18%), followed by Gram-negative bacteria and mixed pathogens (21.82% each), and fungi (18.18%). The most common organisms were *Streptococcus, Staphylococcus, Pseudomonas, Aspergillus*, and *Candida*, accounting for 56.45% of isolates. Pars plana vitrectomy was done for 62.87% of patients, intravitreal and other antibiotic therapy in 23.27%, and primary enucleation/evisceration in 10.89%. The final outcomes and best corrected visual acuities were: anophthalmia 11.86%, no light perception/no dazzle 27.84%, light perception 8.76%, hand motions 24.23%, counting fingers 5.15%, 3/200 to 20/50 12.89%, and 20/40 to 20/20 9.28%.

**Conclusions:**

There was a higher proportion of post-traumatic endophthalmitis cases compared to traditional estimates but consistent with studies from China and Thailand. The majority of these cases involved younger children as well as young to middle-aged males engaged in carpentry and construction work, implying a need for increased public health awareness and strengthening of childcare and workplace safety policies. Our microbiologic profile showed a lower proportion of Gram-positive infections and a higher proportion of mixed pathogen infections compared to other studies. There was also a higher proportion of fungi associated with post-operative and keratitis-induced endophthalmitis. The best outcomes were seen in acute post-operative and bleb-associated endophthalmitis, and the worst outcomes in endogenous and keratitis-induced endophthalmitis. Visual outcomes were poorer compared to other Western and Asian countries, with only 21.7% of patients improving from presentation.

## Introduction

Endophthalmitis is a rare but sight-threatening disease characterized by marked progressive inflammation of the vitreous and/or aqueous humors, usually due to an intraocular bacterial, fungal, parasitic or rarely, viral infection [1–3]. It is traditionally classified according to etiology as post-operative, post-traumatic, endogenous, or keratitis-induced. Other ways of classification are by the organism(s) involved and by the clinical course (acute or chronic/delayed-onset) [2].

In many developed countries, post-operative cases make up the majority of endophthalmitis [4]. Acute-onset post-operative endophthalmitis is defined as occurring within six weeks of ocular surgery, while chronic or delayed-onset occurs past this period. Most patients with the acute-onset type present within seven days. Cataract surgeries, one of the most common eye operations performed worldwide, are responsible for the majority of acute cases with reported incidences ranging from 0.03% to 0.2% [1]. Chronic post-operative endophthalmitis occurring after cataract surgery is less common, with one study reporting an incidence of 0.02% [5]. Another review showed that it only accounted for 7.2% of culture-proven post-operative endophthalmitis cases [6]. Chronic post-operative endophthalmitis progresses slowly and may present with only mild inflammation [1].

Bleb-associated and suture-related endophthalmitis can also be classified as post-operative endophthalmitis. Bleb-associated endophthalmitis is usually seen in the late (more than 4 weeks) post-surgical period following a trabeculectomy, but may also present acutely. The time from surgery to presentation can range from 1.5 to 7 years. Incidences from 0.17% to 13.2% have been reported [1]. The risk of endophthalmitis is estimated to be 1.3% per patient-year [3]. Blebitis should also be ruled out in these cases [1].

Relatively few cases of corneal suture-related endophthalmitis have been reported in the literature. One retrospective consecutive case series showed only six endophthalmitis cases associated with culture-proven corneal suture infections within a 15-year period [7].

Post-traumatic endophthalmitis is a significant complication that can follow penetrating eye trauma. Incidences from 0% to 12% have been reported, increasing to as much as 60% in the presence of an intraocular foreign body (IOFB) [1, 2]. Early surgical repair and prophylactic systemic antibiotics may reduce this incidence to < 1% [3]. It is estimated that around 25% of endophthalmitis cases are due to ocular trauma [8].

Endogenous endophthalmitis is uncommon, representing an estimated 2% to 16% of all reported endophthalmitis cases [1]. The majority of patients are adults, with pediatric cases accounting for only 0.1% to 4% [2]. There may either be evident systemic sepsis, or a multiorgan subclinical or localized infection. In the latter, transient bacteremia or fungemia may explain the spread of the pathogen to the eye [2].

Infectious keratitis does not usually progress to endophthalmitis. A large retrospective study showed that only 0.5% of eyes with suspected infectious keratitis advanced to culture-proven endophthalmitis [9]. However, it is usually difficult to distinguish keratitis from endophthalmitis because they both present with pain, significant visual loss, hypopyon, and poor view of the posterior segment.

To the authors’ knowledge, there has only been one local study on endophthalmitis to date. De Sagun-Bella and Santos analyzed 114 endophthalmitis cases seen from 2005 to 2015 by the Surgical Retina service of the Department of Ophthalmology and Visual Sciences of the Philippine General Hospital (DOVS-PGH), the largest tertiary referral hospital in the Philippines [10]. They reported that post-traumatic cases were the most common type (64.9%), followed by post-surgical (24.5%) and endogenous cases (10.5%). Management was as follows: 81.5% underwent pars plana vitrectomy (PPV), 11.4% were enucleated, and 7.0% of eyes were managed conservatively as these were in beginning phthisis. The best corrected visual acuities (BCVA) at 90 days post-operatively was at most 20/400. However, patients seen and managed by the External Disease and Cornea service alone, to which all endophthalmitis patients are initially referred, were not included in the study population. There was also a lack of keratitis-induced cases in their study. In addition, some subgroups were not analyzed separately, such as acute from chronic post-operative cases, and bleb-associated cases from other post-operative cases. Microbial profile analysis was also limited to reporting the top causative organisms. Lastly, the reported visual outcomes do not represent the BCVA past 90 days, when they might have improved further.

Treatment of endophthalmitis is challenging, and identification of risk factors and causative organisms is part of successful management. At present, there is still limited information available regarding endophthalmitis cases in the Philippines. There are also no reported microbial profiles and long-term visual outcomes to date. This study aims: [1] to determine the incidence and etiology of endophthalmitis over a ten-year period, and for each type, the following: associated risk factors, causative pathogens, management, and visual outcomes; and [2] to compare the results of the study to existing literature.

## Methods

This study is a single center, retrospective review of all endophthalmitis cases diagnosed at the DOVS-PGH in Manila, Philippines, over a ten-year period from January 1, 2007 to December 31, 2016. Cases were classified according to their etiology, and data pertaining to history, associated risk factors, isolated microorganisms, management, and final visual outcomes for each patient were reviewed and analyzed. The study was conducted according to the tenets of the Declaration of Helsinki and was approved by the Research Ethics Board of the University of the Philippines – Manila.

## Results

A total of 223 endophthalmitis cases within the 10-year audit period were retrieved, amounting to an average of 22 new endophthalmitis cases seen annually. Two hundred and two endophthalmitis cases were analyzed in this review after exclusion of 21 cases due to incomplete data.

### Demographic data

The cases were classified into seven types of endophthalmitis according to etiology and, for post-operative cases, their course (Table 1). The most common was post-traumatic, accounting for more than half of all cases (55.94%). This was followed by endogenous endophthalmitis at 14.36% and acute post-operative endophthalmitis at 10.40%. Among 46 patients with preceding surgery (acute and chronic post-operative, bleb-associated, and suture-associated cases), 22 (47.83%) had been performed at the DOVS-PGH. The male to female ratio was 2.5:1 overall, and was highest at 6.5:1 in the post-traumatic group. The average duration of follow-up was 309 days.

**Table 1:** Classification and demographic data of endophthalmitis cases (*n* = 202). Post-traumatic cases made up more than half of the population, and were associated with younger age and a higher male to female ratio

Type of endophthalmitis	Number of patients	Average age (years)	Gender		Average follow-up (days)
			Male	Female	
Post-traumatic	113 (55.94%)	24.2	86.73%	13.27%	248
Endogenous	29 (14.36%)	37.4	51.72%	48.28%	371
Acute post-operative	21 (10.40%)	60.8	47.62%	52.38%	607
Keratitis-induced	14 (6.93%)	53.6	78.57%	21.43%	266
Chronic post-operative	11 (5.44%)	61.5	27.27%	72.73%	375
Bleb-associated	8 (3.96%)	50.3	50.00%	50.00%	323
Suture-associated	6 (2.97%)	39.7	50.00%	50.00%	87

**Fig. 1:**
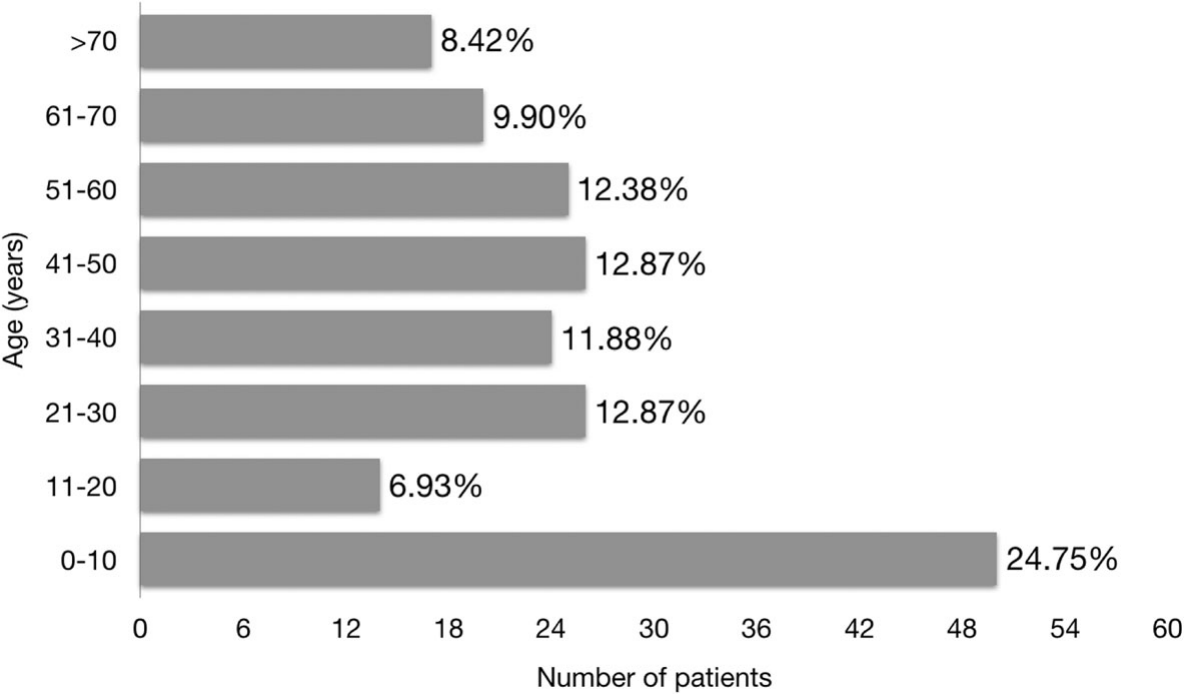
Age distribution of endophthalmitis cases (*n* = 202). The largest age group affected was 10 years old and below, constituting one-fourth of the cases, while at least half of the patients were within the working age group

The mean patient age in our study was 35.5 years. The most commonly affected age group was 0–10 years, which accounted for one-fourth of all cases (Fig. 1). Patients of working age constituted at least half of the population.

**Fig. 2:**
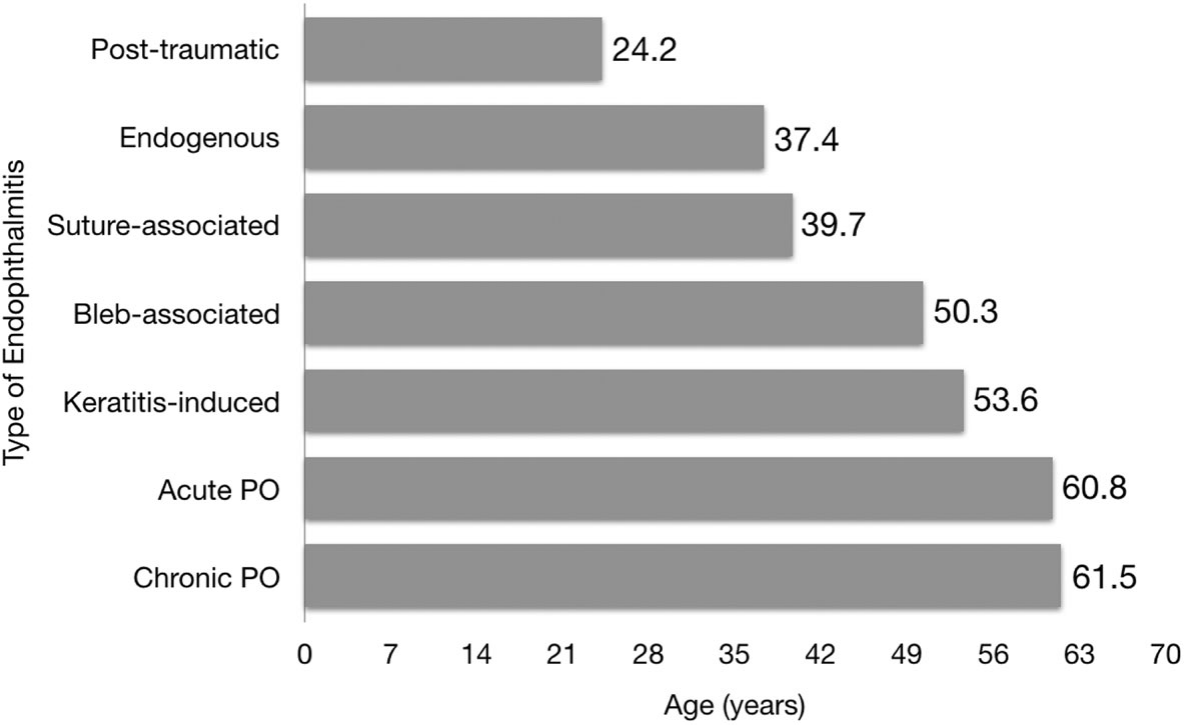
Mean age by cause of endophthalmitis (*n* = 202). Post-traumatic cases were of younger age on average (24.2 years) compared to acute and chronic post-operative cases (60.8 and 61.5 years, respectively), most of whom had undergone cataract surgery. PO = post-operative

Figure 2 shows the groups according to mean age. The post-traumatic endophthalmitis group had the youngest mean age of 24.2 years, while acute and chronic post-operative cases were at the other end of the spectrum with mean ages of 60.8 and 61.5 years, respectively. This is due to the majority of the latter groups having preceding cataract surgeries.

### Culture results

**Fig. 3:**
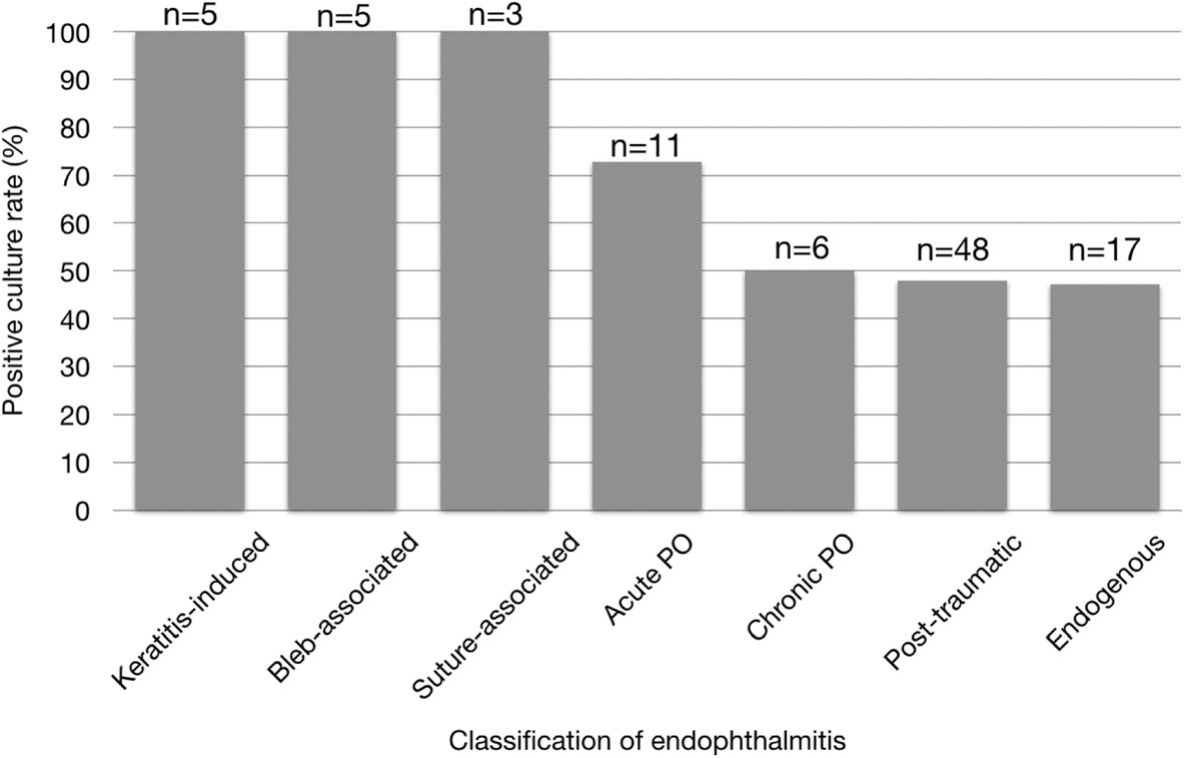
Positive rates of microbial cultures among the different groups (*n* = 95). Albeit fewer, keratitis-induced, bleb-associated, and suture-associated cases had high culture positivity rates, while only around half of chronic post-operative, post-traumatic, and endogenous cases yielded positive cultures. PO = post-operative

Most of the cases (170; 84.16%) had ocular samples (vitreous, aqueous, intraocular lens (IOL), IOFB, posterior capsule, corneal scraping, conjunctival swab) sent for microbial analysis. Of the 95 cases with retrieved culture results, 55 (57.89%) were positive and 40 (42.11%) were negative. Culture positivity among the different groups is shown in  Fig. 3. The highest rates were seen in keratitis-induced, bleb-associated and suture-associated endophthalmitis, while the lowest rates were seen in chronic post-operative, post-traumatic, and endogenous endophthalmitis.

**Fig. 4:**
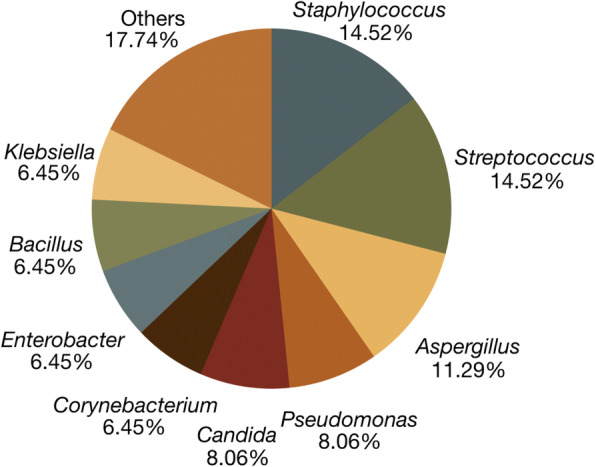
Most common organisms among microbial isolates identified (*n* = 62). The most prevalent organisms were *Staphylococcus* and *Streptococcus* species. *Aspergillus* and *Pseudomonas* in addition to these made up half of all organisms identified by culture

Gram-positive organisms were responsible for 38.18% of culture-positive cases, Gram-negative and mixed pathogens each for 21.82%, and fungi for 18.18%. Among 62 microbial isolates obtained from the 55 culture-proven cases, the most common organisms were *Staphylococcus* and *Streptococcus* (14.5% each) (Fig. 4). Other common organisms were *Aspergillus* (11.3%), *Pseudomonas*, and *Candida* (8.1% each). The aforementioned organisms in total made up 56.45% of isolates.

Table 2 summarizes the profile of causative microorganisms among the different types of endophthalmitis. Gram-positive organisms were more predominant in post-traumatic and endogenous infections, while there was an even distribution of organisms in acute post-operative cases. Fungi were more predominant in keratitis-induced and chronic post-operative endophthalmitis. Gram-positive bacteria and mixed pathogens were mostly seen in bleb-associated endophthalmitis, while Gram-positive, Gram-negative, and mixed infections were equally associated with suture-related endophthalmitis.

**Table 2:** Distribution of causative pathogens among the different groups (*n* = 55). Mixed cultures yielded a combination of Gram-positive, Gram-negative, and/or fungal isolates. Gram-positive bacteria were predominant in the post-traumatic and endogenous groups, fungi in the chronic post-operative and keratitis groups, and mixed pathogens in the bleb-associated group

Pathogen	Post-traumatic (***n*** = 23)	Endogenous (***n*** = 8)	Acute PO (***n*** = 8)	Chronic PO (***n*** = 3)	Keratitis (***n*** = 5)	Bleb (***n*** = 5)	Suture (***n*** = 3)
**Gram-positive bacteria**	52.2%	37.5%	25.0%	0%	20.0%	40.0%	33.3%
**Gram-negative bacteria**	30.4%	12.5%	25.0%	0%	20.0%	0%	33.3%
**Fungi**	4.3%	25.0%	25.0%	66.67%	40.0%	20.0%	0%
**Mixed pathogens**	13.0%	25.0%	25.0%	33.33%	20.0%	40.0%	33.3%

PO = post-operative

### Management

Overall, 62.87% of patients underwent PPV, 23.27% received intravitreal and other antibiotics, 10.89% underwent primary enucleation or evisceration, and 2.97% underwent other surgeries without vitrectomy. Among the different groups, keratitis-induced cases had the highest rates of primary evisceration and enucleation at 28.6% (Table 3). PPV was done for most cases except in endogenous and keratitis-induced endophthalmitis where medical management was the mainstay. The  average time to surgery (excluding evisceration and enucleation) was 3.3 days. For post-traumatic cases, the average time was 2.7 days.

**Table 3:** Management of endophthalmitis cases among the different groups (*n* = 202). The majority of patients underwent PPV except in the endogenous and keratitis groups where management was mostly medical. Keratitis-induced endophthalmitis was associated with the highest rates of primary evisceration and enucleation

Management	Post-traumatic (***n*** = 113)	Endogenous (***n*** = 29)	Acute PO (***n*** = 21)	Chronic PO (***n*** = 11)	Keratitis (***n*** = 14)	Bleb (***n*** = 8)	Suture (***n*** = 6)
**Pars plana vitrectomy +/− other procedures**	77.0%	20.7%	71.4%	54.5%	21.4%	75.0%	66.7%
**Intravitreal and other antibiotics only**	7.96%	69.0%	19.0%	36.4%	50.0%	25.0%	16.7%
**Primary Evisceration or enucleation**	12.4%	10.3%	0%	0%	28.6%	0%	16.7%

PO = post-operative

### Final outcomes

**Fig. 5:**
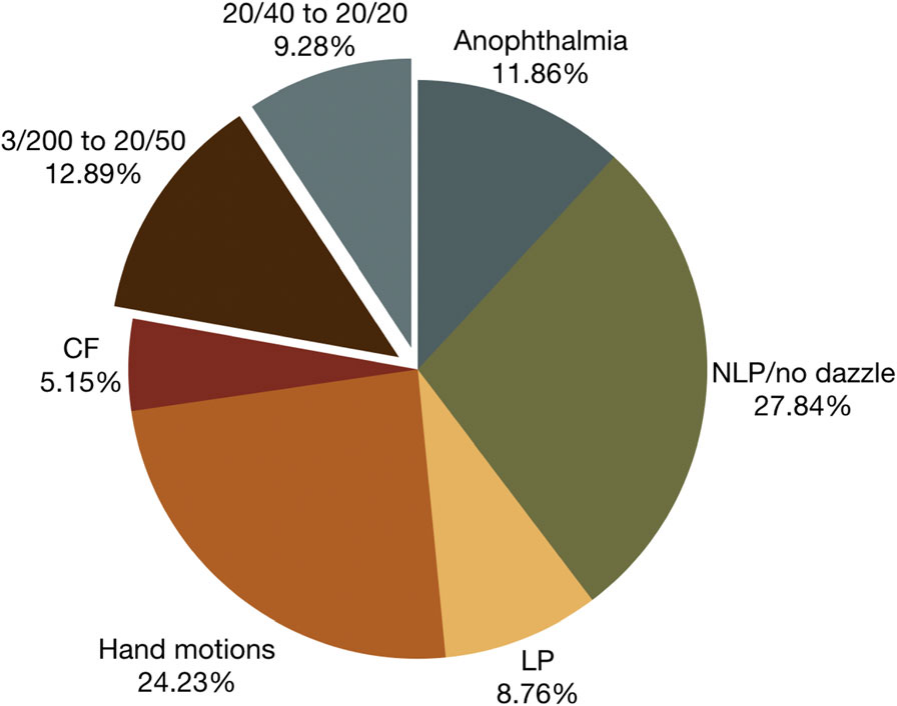
Final best corrected visual acuities of patients at discharge (*n* = 194), exluding eight pre-verbal children with vision better than NLP or no dazzle. Forty percent of patients either lost the globe or lost all vision. Only 22% of patients were left with useful vision. NLP = no light perception; LP = light perception; CF = counting fingers

Around three-fourths of all patients had no to poor vision at discharge (Fig. 5). Almost 12% of patients became anophthalmic, while 12.89% recovered to 3/200 to 20/50, and 9.28% recovered to at least 20/40 BCVA. Overall, only 21.7% patients had improved BCVA from presentation. Visual outcomes for eight young pre-verbal children with vision better than no light perception (NLP) or no dazzle were not included in this analysis.

The  endogenous endophthalmitis group had the highest proportion of patients who worsened to NLP or no dazzle (Table 4). Acute and chronic post-operative endophthalmitis patients had the greatest rates of recovery to at least 20/40 BCVA.

**Table 4:** Final BCVA among the different groups (*n* = 194), excluding eight pre-verbal children with vision better than NLP or no dazzle. The acute post-operative and bleb-associated groups had the best outcomes, while the endogenous and keratitis groups had the worst outcomes

Outcome	Post-traumatic (***n*** = 106)	Endogenous (***n*** = 28)	Acute PO (***n*** = 21)	Chronic PO (***n*** = 11)	Keratitis (***n*** = 14)	Bleb (***n*** = 8)	Suture (***n*** = 6)
**Anophthalmia**	13.2%	10.7%	4.8%	0%	28.6%	0%	16.7%
**NLP/no dazzle**	23.6%	60.7%	19.0%	18.2%	28.6%	25.0%	0%
**LP**	9.4%	3.6%	14.3%	9.1%	7.1%	12.5%	0%
**HM**	28.3%	10.7%	19.0%	27.3%	21.4%	12.5%	50.0%
**Counting Fingers**	4.7%	0%	4.8%	18.2%	0%	12.5%	16.7%
**3/200 to 20/50**	9.4%	14.3%	23.8%	9.1%9.1%	7.1%	37.5%	16.7%
**20/40 to 20/20**	11.3%	0%	14.3%	18.2%	7.1%	0%	0%

NLP = no light perception, LP = light perception, HM = hand motions

### Post-traumatic endophthalmitis

**Fig. 6:**
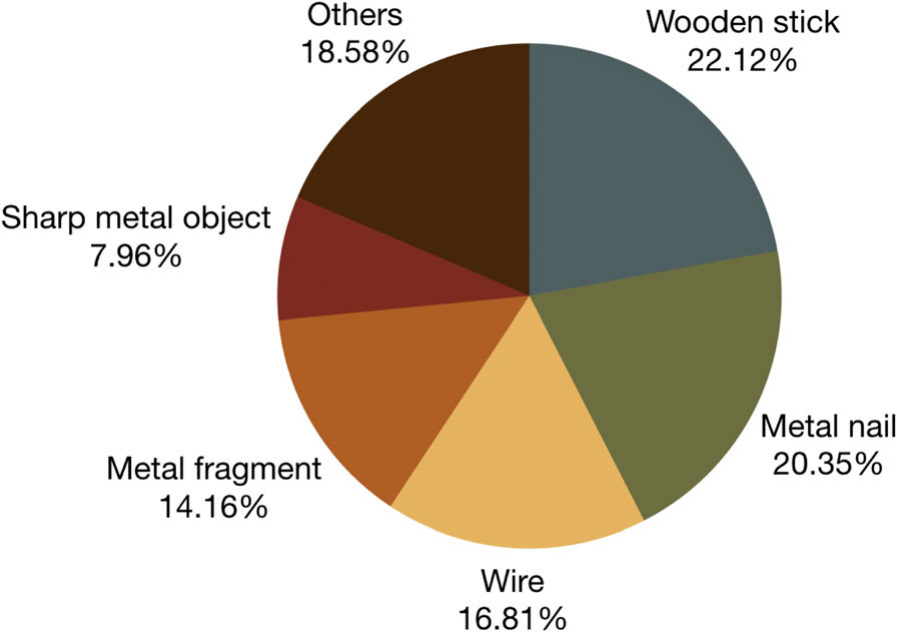
Offending agents in post-traumatic endophthalmitis cases (*n* = 113). Wooden sticks were the most common traumatic objects and were often associated with pediatric cases. Metal nails, wires, and metal fragments were also among the most frequently identified offending agents

More than two thirds of the 113 patients were male (86.73%) (Table 1). The largest age group affected was 0–9 years old (31.86%), with ocular trauma usually occurring due to lack of supervision and failure to remove sharp or pointed objects from the child’s vicinity. Injury was most frequently caused by barbecue sticks or *walis tingting* (native whisk broom) (22.12%) (Fig. 6). Other common offending agents were metal nails (20.35%), wires (16.81%), and metal fragments (14.16%). At least half of post-traumatic cases was due to the use of industrial tools. Although most of these events were due to lack of eye protection during carpentry and construction work, some still occurred despite protective eyewear having been worn.

Associated factors for infection noted in our patients include the following: delayed consult and increased time to vitrectomy, posterior capsule rupture and vitreous loss, presence of an IOFB and/or retinal detachment, and preoperative topical steroid use. Delay in consult was usually due to occurrence of trauma in rural areas, or primary repair without vitrectomy done previously at a different institution. Preoperatively, retinal detachment was seen in 15.93% of patients, while 13.27% was documented pre- or intraoperatively to have an IOFB. At least 17 patients (15.04%) had prior topical steroid use, four of whom had undergone primary repair elsewhere.

Among 23 culture-positive cases, bacteria represented 82.6%, mixed pathogens 13.0%, and fungi only 4.3% (Table 2). Gram-positive bacteria were more commonly isolated than Gram-negative bacteria (52.2% and 30.4%, respectively). Among the most common organisms were *Streptococcus, Staphylococcus, Enterobacter,* and *Aspergillus*. Phthisis developed in 26 cases (26.80%, *n* = 97), while two cases were already in phthisis because of late consult. The average age of these patients was 20.3 years, 53.5% of whom were pediatric cases.

### Endogenous endophthalmitis

Diabetes mellitus, dental cavities, urinary tract infection, lung disease, and sepsis were the most common systemic co-morbidities among the 29 patients with endogenous endophthalmitis (Table 5). Some patients had more than one risk factor, including all five patients with sepsis who also had diabetes. Only 10.34% of endogenous endophthalmitis cases were bilateral, with all having known co-morbidities. However, no predisposing factor could be identified in 17.24% of patients.

**Table 5:** Identified risk factors for endogenous endophthalmitis (*n* = 29). Diabetes mellitus, dental cavities, and urinary tract infections were the most commonly identified risk factors

Associated Factor	Number of Patients (%)
Diabetes mellitus	10 (34.48%)
Dental cavities	8 (27.59%)
Urinary tract infection	8 (27.59%)
Lung disease	6 (20.69%)
Sepsis	5 (17.24%)
Non-ocular mass	2 (6.90%)
Chronic kidney disease	1 (3.45%)
Cellulitis	1 (3.45%)

The most common organisms retrieved from eight culture-positive cases were *Bacillus, Corynebacterium,* and *Aspergillus*. Four patients presented with panophthalmitis, three of whom were known diabetics. Three of these patients underwent evisceration/enucleation while one developed phthisis bulbi. Phthisis developed in a total of nine out of 25 patients (36.0%), excluding one case with phthisis at consult. Six of these patients had been medically managed only, while two had undergone surgery.

### Acute post-operative endophthalmitis

Most of the 21 patients (71.43%) had undergone cataract surgery, with phacoemulsification accounting for more than half of all cases (57.14%). The ratio of phacoemulsification to extracapsular cataract extraction (ECCE) procedures was 13:1, while one case remained unspecified. Among these patients, ten had an intact posterior capsule with posterior chamber IOL, two patients had intraoperative vitreous loss, while three patients had incomplete documentation. PPV as the preceding surgery accounted for 19.05% of cases, half of which were done for phacoemulsification complications.

Vitreous loss was documented in only 27.78% of all patients (excluding those with incomplete documentation). Risk factors in this group were multiple consecutive procedures for complicated cataract surgeries, poor patient hygiene, and suture dehiscence following anterior chamber IOL implantation. Associated factors among those without a history of vitreous loss include scleral perforating injury from a metal nail, use of a re-sterilized phacoemulsification cassette, and persistent intraoperative iris prolapse. Diabetes was documented in 23.81% of patients in this group. No risk factor however could be identified in 19.05% of patients.

There was an equal prevalence of Gram-positive, Gram-negative, fungal, and mixed pathogens among the eight cases which yielded positive cultures (Table 2). The most commonly isolated organisms were *Candida* and *Pseudomonas*. PPV with IOL explant or other procedures were done for most patients (Table 3). Two of four patients regained vision with medical management only. Secondary evisceration was done for one patient, after initial PPV and IOL explant; this was the only patient in the group who became anophthalmic.

### Chronic post-operative endophthalmitis

Most patients who had chronic infection developed symptoms within five months from surgery. Only six of 11 (54.55%) patients had previous cataract surgery, slightly less than that in the acute group. The numbers of phacoemulsification and ECCE procedures were comparable (two and three, respectively). Three had an intact posterior capsule with posterior chamber IOL implant, one had intraoperative vitreous loss, while two had incomplete documentation. Of the three patients with an intact posterior capsule, two had no identified risk factor while one was documented to have a loose suture over the main wound, possibly causing a slow leak.

Similar to the acute group, 18.18% of patients developed symptoms after PPV. Indications for surgery had been diabetic retinopathy and epiretinal membrane. In the latter case, the patient’s anterior chamber IOL haptic slowly eroded through the sclera and endophthalmitis developed 4.5 years post-operatively. These two patients were the only known diabetics in the group (18.18%).

A history of vitreous loss was noted in 55.56% of all patients (excluding those with incomplete documentation). Other associated factors include post-keratoplasty for a ruptured *Staphylococcus epidermidis* ulcer (which developed *Cladosporium* endophthalmitis), previous retina surgery for an IOFB, and blepharitis.

Of the three cases which yielded positive cultures, two were fungal and one had mixed pathogens (Gram-positive cocci and Gram-negative bacilli) (Table 2). Only one isolate, however, was identified (*Cladosporium* sp.) as the rest were still for speciation. The main treatment was vitrectomy with or without IOL explant. In addition, 36.4% of patients were managed medically, with half of these patients regaining vision (Table 3).

### Keratitis-induced endophthalmitis

Keratitis-induced endophthalmitis was associated with foreign body trauma, ophthalmic laser procedures and subsequent steroid use, and recent post-keratoplasty for fungal keratitis. Among four patients (28.57%) who had recently used topical steroids, at least one had not been on any topical antibiotic. Two of the 14 patients presented with a perforated corneal ulcer; one patient had undergone a repeat penetrating keratoplasty (PKP) nine years ago and had recent topical steroid use, while the other had no identifiable risk factor. Half of the patients in this group had no recent history of trauma, ocular surgery, or steroid use. Among these were an adult patient with old measles leukoma and a patient with diabetes.

Among the five cases which had positive cultures, two were fungal (40.0%), one yielded Gram-positive bacteria (20.0%), one yielded Gram-negative bacteria (20.0%), and one had mixed pathogens (Gram-positive and Gram-negative organisms) (20.0%) (Table 2). Organisms identified were *Pseudomonas aeruginosa*, *Enterobacter, Klebsiella, Fusarium, Aspergillus,* and *Streptococcus pneumoniae*. Half of the patients were medically managed, with one case improving to 20/70 BCVA. Almost a third (28.6%) of patients underwent enucleation or evisceration, the highest rate in our study. Among 21.4% of patients who underwent PPV and/or other procedures, vitrectomy with PKP was done in 14.3% (two cases), both of which had visual outcomes of hand motions.

### Bleb-associated endophthalmitis

Almost all of the eight cases developed 3–11 years post-trabeculectomy. Only one patient developed endophthalmitis less than 1 year post-operatively, after resuturing for an early bleb leak. Excluding this, the average duration from trabeculectomy to infection was 7.4 years. Three cases had documented use of mitomycin-C during trabeculectomy, while antimetabolite use in the other cases was undocumented. None of the patients in our study were known diabetics, while two cases (25.0%) had no identifiable risk factor.

A young male with poor history of follow-up was the only patient in this group who developed bilateral consecutive bleb-associated endophthalmitis. Trabeculectomies with mitomycin-C for bilateral juvenile open angle glaucoma were done separately four years ago. The risk factor for this patient for the first eye was poor follow-up and discontinuation of medications, while none was identified for the second eye.

Five cases were culture-positive (40.0% Gram-positive bacteria, 40.0% mixed pathogens, and 20.0% fungi) (Table 2). Isolated microorganisms included *Staphylococcus aureus* and *epidermidis, Streptococcus viridans,* and *Aspergillus niger*. Most of the patients underwent vitrectomy with or without IOL explant (75.00%) (Table 3). One of the two patients who were managed medically improved.

### Suture-associated endophthalmitis

The six patients in this group either had preceding removal of sutures, retained long-term sutures, exposed suture knots, or loose sutures. The sutures had been in place from a range of two months to two years. Two patients (retained and loose suture cases) were using topical steroids at the time; the latter did not have concurrent topical antibiotic cover. One patient with a two-year retained suture had washed her eye with tap water for foreign body sensation. Among two patients who had undergone removal of sutures, one was an uncooperative child and one had only started the topical antibiotic one day later. Only one patient was documented to have diabetes, and had only been diagnosed at our hospital upon workup. Four cases were post-cataract surgery (two ECCE and two unspecified), one of whom had intraoperative vitreous loss. One case had undergone phacoemulsification and trabeculectomy, and one case was a secondary IOL implantation.

The  three cases with retrieved results had positive cultures with equal prevalence of Gram-positive, Gram-negative, and mixed pathogens (Gram-negative bacteria and fungal) (Table 2). Organisms identified were *Staphylococcus* sp*., Proteus mirabilis*, and a mixed infection of *Fusarium solani* and *Acinetobacter lwoffii*. Most cases underwent PPV with or without IOL explant (Table 3).

## Discussion

Our population is almost double that of De Sagun-Bella and Santos in their pilot study [10]. In contrast to previous reports from developed countries, including the United States, Canada, New Zealand, and the United Kingdom, where most cases of endophthalmitis are due to intraocular surgery [6, 8, 11–13], post-traumatic endophthalmitis accounted for the majority of cases (55.94%) in our study. Our findings however are consistent with reviews done in China and Thailand, where the major etiology of infectious endophthalmitis cases was ocular trauma (Table 6) [4, 16, 17]. Higher rates of ocular injury associated with industrial and agricultural activities in developing countries could be responsible for the dissimilarity of results among the studies [4]. A study conducted in India on the other hand showed comparable rates of post-traumatic and post-operative endophthalmitis [18]. Our respective proportions of post-operative and endogenous endophthalmitis were comparable to other developing countries; however, our proportion of keratitis-induced infections was twice as high compared to studies done in China and Thailand (Table 6).

**Table 6:** Distribution of types of endophthalmitis compared to other countries. Asia-Pacific countries showed a higher proportion of post-traumatic cases compared to Canada and the UK

Type of endophthalmitis	Canada [14] (***n*** = 114)	UK [11] (***n*** = 47)	New Zealand [15] (***n*** = 106)	Our study (***n*** = 202)	China [4, 16] (***n*** = 330; 1593)	Thailand [17] (***n*** = 420)	Eastern India [18] (***n*** = 107)
**Post-traumatic**	12%	2%	18.9%	55.94%	58.5%, 82.6%	43.1%	40.2%
Post-operative*	47%	92%	58.5%	22.77%	20.3%, 6.9%	32.2%	43%
**Endogenous**	41%	6%	16%	14.36%	18.5%, 7.8%	3.3%	16.8%
**Keratitis-induced**	–	–	–	6.93%	2.7%, 2.7%	3.1%	–

*Includes acute and chronic post-operative, and bleb- and suture-associated endophthalmitis

Post-traumatic endophthalmitis cases were associated with younger age, and acute and chronic post-operative cases with older age as most of the latter had undergone cataract surgeries (Table 1). Male patients represented the majority of our cases, similar to studies in other developing countries where figures ranged from 66.0% to 79.5% [16]. This could be because our hospital receives patients of relatively lower economic status and a large number of these cases are industry-related post-traumatic endophthalmitis. Gender-based behavior and engagement of males in higher risk activities may also be contributory reasons [4]. In contrast, there appears to be no gender predilection in developed countries, with a study from Canada reporting 45.6% males [14].

Our culture-positive rate of 57.89% is higher compared to the other Asian countries and the USA, but less than that in a Canadian study (Table 7). The spectrum of organisms partly depends on the geographical location of the patient. Table 8 compares our microbiologic profile to similar retrospective studies. Compared to other developing Asian countries, our study shows a lower proportion of Gram-positive bacteria, less fungal infections compared to India and southern China, and a higher proportion of mixed pathogen infections. This disparity may also be due to more difficult cases being referred to our institution. Some of these patients had been treated for infection before being referred, thereby also potentially altering the initial microbial profile.

**Table 7:** Culture-positivity compared to other studies. Our culture-positivity rate was higher compared to other Asian countries as well as the USA, but lower than in Canada

Country	Culture-positive rates
**Canada** [14] **(*n*** **= 88)**	76%
**USA** [12] **(*n*** **= 67)**	44.4%
**Our study (*n*** **= 95)**	57.89%
**Eastern India** [18] **(*n*** **= 107)**	42.1%
**Southern India** [19] **(*n*** **= 955)**	44.4%
**Southern China** [4] **(*n*** **= 330)**	31.8%
**Western China** [16] **(*n*** **= 670)**	39.7%
**Thailand** [17] **(*n*** **= 420)**	29.1%

**Table 8:** Microbiologic profile compared to other countries. Our study showed a lower proportion of Gram-positive bacteria, less fungal infections compared to India and southern China, and a higher proportion of mixed pathogen infections

Organism	Canada [14] (***n*** = 67)	Our study (***n*** = 55)	Southern China [4] (***n*** = 105)	Western China [16] (***n*** = 266)	Thailand [17] (***n*** = 122)	Eastern India [18] (***n*** = 45)
**Gram-positive**	65.7%	38.18%	45.7%	66.5%	52.5%	42.2%
**Gram-negative**	6.0%	21.82%	28.6%	24.1%	28.7%	26.7%
**Fungal**	16.4%	18.18%	23.8%	4.1%	6.56%	26.7%
**Mixed pathogens**	11.9%	21.82%	1.9%	5.3%	12.3%	4.4%

A similar study conducted in western China showed Gram-positive bacteria to be the most prevalent across all types of endophthalmitis [16]. In our study, this was only true for post-traumatic and endogenous infections. Post-operative and keratitis-induced cases had a relatively high proportion of fungal infections (Table 9). Our study results are consistent with the literature wherein *Staphylococcus* and *Streptococcus* are the most common causative organisms (Table 10) [2, 20]. *Aspergillus* and *Pseudomonas* were similarly prevalent in other Asian countries.

**Table 9:** Causative microorganisms among the different types of endophthalmitis in our study (*n* = 55). Post-traumatic and endogenous cases had a higher prevalence of Gram-positive bacteria, while post-operative and keratitis-induced cases had a relatively high proportion of fungal infections

Pathogen	Post-traumatic (***n*** = 23)	Post-operative* (***n*** = 19)	Endogenous (***n*** = 8)	Keratitis-induced (***n*** = 5)
**Gram-positive**	52.2%	26.3%	37.5%	20.0%
**Gram-negative**	30.4%	15.8%	12.5%	20.0%
**Fungal**	4.3%	26.3%	25.0%	40.0%
**Mixed pathogens**	13.0%	31.6%	25.0%	20%

*Includes acute and chronic post-operative, and bleb- and suture-associated endophthalmitis

**Table 10:** Most common organisms compared to other countries. The most prevalent organisms were *Staphylococcus* and *Streptococcus*

Country	Most common isolates
**Canada** [14] **(*n*** **= 59)**	*Staphylococcus, Streptococcus, Candida, Enterococcus*
**USA** [21] **(*n*** **= 313)**	*Staphylococcus, Streptococcus, Propionibacterium acnes*
**Australia** [6] **(*n*** **= 229)**	*Staphylococcus, Streptococcus, Candida*
**Our study (*n*** **= 62)**	*Staphylococcus, Streptococcus, Aspergillus, Pseudomonas, Candida*
**Southern China** [4] **(*n*** **= 105)**	*Staphylococcus, Aspergillus, Fusarium, Pseudomonas*
**Thailand** [17] **(*n*** **= 107)**	*Staphylococcus, Streptococcus, Pseudomonas, Bacillus*
**Eastern India** [18] **(*n*** **= 34)**	*Pseudomonas, Streptococcus, Staphylococcus, Bacillus, Aspergillus, Candida*
**Southern India** [19] **(*n*** **= 46)**	*Staphylococcus, Streptococcus, Pseudomonas, Aspergillus*

In terms of management, our study showed a higher frequency of PPV and a lower frequency of primary enucleation and evisceration compared to other Asian countries (Table 11). However, only 21.7% of our patients had an improvement in BCVA from presentation. This was one-third of the rates seen in Canada, USA, and Thailand, and was slightly lower than that reported in China (Table 12).

**Table 11:** Management of endophthalmitis cases compared to other studies. Our study showed a higher frequency of PPV and a lower frequency of primary enucleation and evisceration compared to other Asian countries

Management	Canada [14] (***n*** = 114)	Our study (***n*** = 202)	Western China [16] (***n*** = 266)	Thailand [17] (***n*** = 122)
**Pars plana vitrectomy**	70.8%	62.87%	51.3%	45.0%
**Topical/intravitreal antibiotics**	15.0%	23.27%	34.8%	20.7%
**Primary enucleation/evisceration**	3.3%	10.89%	13.9%	17.9%

**Table 12:** Final visual outcomes compared to other countries. Only 21.7% of patients in our study had improved BCVA upon discharge

Country	Patients with improved BCVA from baseline (%)
**Canada** [14] **(*n*** **= 114)**	60%
**USA** [12] **(*n*** **= 67)**	61%
**Our study (*n*** **= 194)**	21.7%
**Western China** [16] **(*n*** **= 1424)**	34.4%
**Thailand** [17] **(*n*** **= 420)**	60.2%

### Post-traumatic endophthalmitis

Compared to post-operative endophthalmitis, a greater variety of organisms are found in post-traumatic endophthalmitis since these are derived from the environment [20]. Bacteria cause around 80–90% of culture-positive post-traumatic endophthalmitis [1]. Worldwide, coagulase-negative *Staphylococcus* (i.e., *S. epidermidis* and *S. saprophyticus*) and *Streptococcus* remain the most common isolates, which was also seen in our study. Other significant organisms are *Pseudomonas*, *Enterobacter,* and *Bacillus*, with prevalence depending on the geographic location [2, 20]. A 20-year retrospective study done in China reported fungi among 17.3% of isolates, with *Aspergillus* also being most commonly identified [22]. Virulent organisms associated with post-traumatic endophthalmitis are *Bacillus cereus* and *Staphylococcus aureus* [2, 8, 20]. In our study, *Bacillus*, *Enterobacter*, and *Klebsiella* were retrieved from eyes that eventually required evisceration or enucleation. 

The majority of patients underwent vitrectomy and repair (Table 3). Patients who underwent PPV generally had better outcomes compared to those who had anterior, core, or no vitrectomy, even if it was only done later after primary repair. Other factors associated with poorer outcomes including phthisis bulbi and anophthalmia were delay in consult, panophthalmitis, mixed pathogen infections, and temporary keratoprosthesis use to facilitate view for PPV. Delayed consult could have entailed increased time to vitrectomy, as well as post-operative steroid use after primary repair but prior to vitrectomy.

Post-traumatic endophthalmitis carries a generally poor visual prognosis. A final vision of NLP was reported in one study to be 22.7%, and hand motions or worse to be 45% [23]. Our study showed poorer visual recovery, with almost three-fourths of patients having final visual outcomes of hand motions or worse (Table 4). Some studies have shown that only 15%–41% of cases recover vision to 20/40 or better [1, 24]. A multicenter prospective study similarly noted a return to visual acuity of at least 20/40 in 41% of cases while 35% progressed to phthisis [23]. In our review, only 11.3% of post-traumatic endophthalmitis cases achieved 20/40 or better (Table 4), while phthisis bulbi developed in 26.80% of cases. Younger age and increased number of surgeries did not appear to be significant risk factors for phthisis.

### Endogenous endophthalmitis

Comorbidities such as diabetes mellitus, malignancy, immunocompromisation, immunosuppressive therapy, intravenous drug use, organ abscess, indwelling catheter, urinary tract infection, organ transplant, end-stage renal or liver disease, and endocarditis can predispose patients to the development of endogenous endophthalmitis. It is highly uncommon for patients with endogenous endophthalmitis to have no risk factors for systemic infection, although there have been reports of endogenous endophthalmitis in healthy, immunocompetent individuals without any apparent loci of extraocular infection [2]. Diabetes in particular has been cited as the most common predisposing condition in this group [14, 25], as in our study (Table 5).

The microorganisms in culture-positive cases vary from study to study and is also affected by geographic location [1]. Some are more likely to be found in certain diseases, such as *Staphylococcus aureus* and *Streptococcus* sp. in endocarditis, and *Klebsiella pneumoniae* in liver abscess. Long-term hospitalization has also been implicated in endophthalmitis from *Candida* sp. (especially *albicans*), and filamentous fungi such as *Aspergillus* [13]. Fungi were the most commonly identified pathogens in several studies, notably *Candida albicans* followed by *Aspergillus*. Endogenous endophthalmitis has also been reported to be caused mainly by Gram-positive bacteria in the west, and by Gram-negative bacteria (specifically *Klebsiella*) in East Asian countries [1]. The majority of the isolates in our study, however, were Gram-positive bacteria. The only Gram-negative organism identified was *Klebsiella* from a diabetic patient who had a urinary tract infection. *Aspergillus* was also one of the most common organisms for this group in our study. Non-ocular cultures were positive in three cases, and in one case the blood culture result correlated with the ocular culture (methicillin-resistant *Staphylococcus aureus*).

In addition to systemic therapy for treating the focus of infection in endogenous endophthalmitis, PPV should be strongly considered if there is no response to initial treatment or if the condition is considered severe and sight-threatening [26]. The majority of our cases received medical management only (69.0%) (Table 3). The case with the best visual outcome in the group (20/50), however, had undergone PPV.

Visual prognosis is often poor in both bacterial and fungal endogenous endophthalmitis [2]. *Aspergillus* species have been associated with the worst final visual outcomes and *Candida* species with the best [1, 27]. Another study reported that a final visual acuity of at least 20/200 was achieved in 80% of eyes with *Candida* isolates, and in only 18% of cases caused by Gram-positive bacteria [28]. In our study, *Aspergillus* was isolated from one of the three cases which required evisceration/enucleation, while *Bacillus* was the only organism associated with better than NLP outcomes. Mixed pathogens were retrieved from 25.0% of cases (Table 2), all of whom had final visual outcomes of NLP or no dazzle.

A meta-analysis which included endogenous endophthalmitis cases from reported that a final visual acuity of 20/200 or better was attained by 41% of eyes, while 19% eventually required enucleation or evisceration [1]. In an East Asian study, only 28% of cases regained good visual acuity [2]. In contrast, only 14.3% of patients in our study achieved at least 3/200 final BCVA, and none improved to 20/40 or better (Table 4). Also, evisceration/enucleation was done in only 10.3% of cases (Table 3). Phthisis bulbi developed in 36.0% of patients, the majority of which had been only managed medically.

### Acute post-operative endophthalmitis

Known risk factors for post-operative endophthalmitis also documented in our study include diabetes mellitus, older age, intraoperative complications, posterior capsular rent, vitreous loss, less experienced surgeons, and wound leak [1]. Posterior capsular rent has been shown to increase the risk of acute endophthalmitis by as much as 8 to 11-fold [31]. Vitreous loss however was documented in only 27.78% of patients in our study.

Among those who underwent cataract surgery in this group, phacoemulsification significantly outnumbered ECCE procedures. This could also be attributed to more phacoemulsification procedures being performed in our institution. It is still debatable whether there is an increased risk of acute endophthalmitis after ECCE compared to phacoemulsification. Some studies have shown a higher incidence after phacoemulsification [5, 31], some studies showed the opposite conclusion [32–34], while others have found no difference between the two surgical techniques [35].

The most commonly reported causative microorganisms in acute post-operative endophthalmitis are coagulase-negative staphylococci, *Staphylococcus aureus*, streptococci, other Gram-positive cocci, including enterococci and mixed bacteria, and Gram-negative bacilli. The usual pathogenesis, contamination of the aqueous humor with surface bacteria during surgery, is reflected in the fact that Gram-positive bacteria cause more than 95% of these cases [3]. However, two studies from India reported that the incidence of Gram-positive and Gram-negative bacteria were approximately equal. There was also a higher incidence of *Pseudomonas aeruginosa* endophthalmitis [2].

Though less common than bacterial endophthalmitis, fungal endophthalmitis can also occur post-cataract surgery with the majority also presenting acutely [2]. In tropical regions such as India, fungi may cause 10-15% and as high as 17-22% of post-operative endophthalmitis cases [1, 3]. Similar to the studies from India, Gram-positive and Gram-negative bacteria were also of equal incidence among the isolates in our study. However, our study showed a higher incidence of fungal endophthalmitis at 27.27%.

The visual outcome is dependent on the causative organism. Eyes with a final visual acuity of worse than 20/200 are reportedly more likely to be associated with *Streptococcus*, while coagulase-negative *Staphylococcus* was more prevalent in eyes that recovered to 20/40 or better [1]. In contrast, one case which worsened to NLP in our study was due to *Staphylococcus lugdunensis*, while 20/40 to 20/20 BCVA was regained in cases from *Blastoschizomyces capitatus*, *Escherichia coli*, and a mixed pathogen case with *Candida albicans*, *Pseudomonas aeruginosa*, and anaerobic Gram-positive cocci.

Another study reported that 50% of eyes with post-cataract endophthalmitis achieved 20/40 vision, while 10% declined to a final visual acuity of 5/200 or worse [3]. In our review, 61.9% deteriorated to counting fingers or worse, while only 14.3% regained at least 20/40 BCVA (Table 4).

### Chronic post-operative endophthalmitis

Aside from blepharitis, risk factors documented in our study were similar to those in the acute post-operative group. Compared to 27.78% of patients in the acute group, 55.56% of patients in this group had a history of vitreous loss. The numbers of phacoemulsification and ECCE surgeries were similar, in contrast to significantly more phacoemulsification than ECCE procedures in the acute group.

*Propionibacterium acnes* is the most common pathogen to be isolated in chronic post-operative endophthalmitis, accounting for 41-63% of culture-proven cases [1]. Other reported bacteria include *Staphylococcus* sp. (especially *epidermidis*), filamentous bacteria (including *Actinomyces* and *Nocardia* sp.), and *Enterococcus faecalis* [2]. Fungi are also significant causative pathogens, having been reported in 16-27% of these cases [1]. In our study, only *Cladosporium* was positively identified as a causative organism. Chronic post-operative endophthalmitis carries a generally better visual prognosis compared to the acute-onset type. One study reported that 50% of chronic cases improved to a final visual acuity of better than 20/40 compared to 27% of acute-onset cases [29]. In our study, however, only 18.2% of cases in the chronic group improved to at least 20/40 BCVA, compared to a slightly lower 14.3% in the acute group.

Another study found that in cases of chronic post-operative endophthalmitis, eyes infected with *P*. *acnes* had better final visual outcomes than fungal cases, where more than 20% of eyes had final vision worse than 20/200 [30]. In our review, both fungal cases similarly had poor outcomes of NLP and hand motions.

### Keratitis-induced endophthalmitis

Known risk factors for keratitis-induced endophthalmitis that were observed in this group include use of topical steroids, older age, diabetes mellitus, foreign body injury, and organic matter exposure, and corneal perforation [9]. One patient with diabetes had recently undergone keratoplasty at another institution for *Fusarium solani *keratitis. Although steroids had been withheld post-operatively, progression to endophthalmitis could be due to the organism's ability to penetrate an intact cornea [9].

In a 15-year retrospective study by Henry et al., fungi were reported to be the most common organisms, followed by Gram-positive bacteria and Gram-negative bacteria [9]. Another 14-year retrospective study however reported Gram-positive bacteria in 36.9% of cases, followed by Gram-negative bacteria in 18.4%, fungi in 10.5%, and no growth or unknown organism in 31.6% [36]. Both studies were conducted in the United States. Among the isolates in our study, in contrast, most common were Gram-negative bacteria, followed equally by Gram-positive bacteria and fungi.

The most common microbial isolates documented by Henry et al. in their study were *Fusarium*, *Candida*, *Streptococcus pneumoniae*, and *Pseudomonas aeruginosa* [9], which were also identified in our study. The recent post-keratoplasty case with *Fusarium solani* eventually underwent evisceration, while NLP outcome was seen in a mixed pathogen case with *Pseudomonas aeruginosa* and *Klebsiella pneumoniae*.

In the same study by Henry et al., a final visual acuity of 20/50 or better was seen in 14% of cases, while patients with visual acuity worse than 5/200 represented 69% of the study population. Thirty-one percent of patients required evisceration or enucleation [9], a rate comparable to that in our study (Table 3). In contrast, 85.7% of our patients declined to counting fingers or worse, and only 7.1% achieved 20/40 or better BCVA (Table 4).

### Bleb-associated endophthalmitis

Risk factors identified in the patients in our study were poor follow-up, early-onset bleb leak, blepharitis, loose suture, intraoperative use of antimetabolites, and recent eye non-penetrating trauma. However, late-onset bleb leak has been more commonly cited as a risk factor compared to early-onset leak [1]. Younger age, also cited as a risk factor for infection, was not observed in our study as the average age of the patients was 50.3 years. Although the use of antimetabolites, specifically mitomycin-C, increases the success rate of trabeculectomies, their use also increases the risk of endophthalmitis 3-fold [1].

The most common pathogens in early bleb-associated endophthalmitis, similar to acute-onset post-operative endophthalmitis, are *Staphylococcus epidermidis* and *Staphyloccocus aureus* [1]. The only early-onset case in our study similarly was culture-positive for *S*. *epidermidis*. Delayed-onset cases on the other hand are usually caused by *Streptococcus* sp. and *Moraxella catarrhalis* [1]. Other gram-negative bacteria (*Pseudomonas aeruginosa*, *Haemophilus influenzae*, and *Serratia* sp.) have been reported as well [2]. Isolates from late-onset infections in our study were *Streptococcus viridans*, *S*. *aureus*, and *Aspergillus niger*.

The final visual outcome in bleb-associated endophthalmitis varies, but the high prevalence of virulent organisms such as *Streptococcus* sp. and Gram-negative bacteria make the prognosis unfavorable. Studies have reported an outcome of no light perception (NLP) in 23% to 35% of cases [1]. Another series noted a return to at least 20/40 vision in 40% of cases while 30% lost all vision in the affected eye [3]. Similarly in our study, 25.0% of patients had a final visual outcome of NLP or no dazzle, while 37.5% recovered 3/200 or better BCVA (Table 4). Among those with positive isolates, the worst visual outcome (LP) was due to *Streptococcus viridans*, while vision was regained in a case due to *Staphylococcus epidermidis*.

Patients with better vision at presentation also have been shown to obtain better visual outcomes. One study reported that 83% of patients with initial visual acuity of better than LP achieved a final visual acuity of better than 20/40, while only 31% of patients with presenting vision of LP were able to attain the same level of improvement [1]. In our study, however, no patient regained at least 20/40 BCVA even among seven patients who presented with better than LP (Table 4).

### Suture-associated endophthalmitis

Associated factors for endophthalmitis in this group were exposed suture knot, topical steroid use without antibiotic cover in a patient who had a loose suture, application of tap water for foreign body sensation, delay in application of topical antibiotic after suture removal, and difficult suture removal in office in an uncooperative child. Although endophthalmitis due to long-term retention of sutures after cataract surgery or penetrating keratoplasty is very rare, the Royal College of Ophthalmologists recommended the removal of corneal sutures within three months following routine extracapsular cataract surgery [7]. Sedation should also be considered for younger children or uncooperative patients.

In the study by Henry et al. which reviewed corneal suture-associated culture-proven endophthalmitis cases over a 15-year period, only six cases were diagnosed with suture-associated endophthalmitis. Five of the isolates were *Streptococcus* sp. while the remaining was a case with *Serratia marcescens* [7]. The organisms identified by culture in our study, however, were *Staphylococcus, Proteus mirabilis*, *Fusarium solani* and *Acinetobacter lwoffii*. The case with *Staphylococcus* was a diabetic patient who presented with a suture abscess and panophthalmitis after cataract surgery (history of vitreous loss was not known), and eventually underwent enucleation. *Proteus* was isolated from the patient who had washed the eye with tap water; this patient had the best outcome in the group of 20/150 BCVA. Henry et al. reported visual outcomes in their study ranging from 20/150 to NLP [7]. In our review, one patient required enucleation, while final BCVA similarly ranged from 20/150 to hand motions (Table 4).

## Conclusions

Post-traumatic endophthalmitis represented a greater proportion of cases compared to traditional estimates but this was consistent with data from studies in China and Thailand. The majority of these cases involved younger children and young to middle-aged males engaged in carpentry and construction work, implying a need for increased public health eye safety awareness and strengthening of childcare and workplace safety policies. There was a lower proportion of Gram-positive infections and a higher proportion of mixed pathogen infections compared to other studies. We also noted a relatively higher proportion of fungi associated with post-operative and keratitis-induced cases. Final visual outcomes are poorer compared to similar studies in Western and Asian countries, with only 21.7% of patients improving from presentation and less than 10% recovering to 20/40 or better. Overall, acute post-operative and bleb-associated endophthalmitis showed the best visual outcomes, while endogenous and keratitis-induced endophthalmitis had the poorest visual outcomes. The results of this study identify the profile of endophthalmitis cases at a tertiary referral hospital in the Philippines.

## Data Availability

The datasets used and/or analysed during the current study are available from the corresponding author on reasonable request.

## Abbreviations

BCVA: Best corrected visual acuity
CF: Counting fingers
DOVS: Department of Ophthalmology and Visual Sciences
ECCE: Extracapsular cataract extraction
HM: Hand motions
IOFB: Intraocular foreign body
IOL: Intraocular lens
LP: Light perception
NLP: No light perception
PGH: Philippine General Hospital
PKP: Penetrating keratoplasty
PO: Post-operative
PPV: Pars plana vitrectomy
